# Synthesis and Characterization of a New Cobalt(II) Complex with 2-(2-Pyridyl)Imino-N-(2-Thiazolin-2-yl)Thiazolidine (PyTT)

**DOI:** 10.1155/S1565363304000184

**Published:** 2004

**Authors:** A. Bernalte-García, F. J. García-Barros, F. J. Higes-Rolando, F. Luna-Giles, M. M. Pacheco-Rodríguez, E. Viñuelas-Zahínos

**Affiliations:** Departamento de Química Inorgánica Facultad de Ciencias Universidad de Extremadura Badajoz 06071 Spain

## Abstract

The compound aquanitrate-кObis[2-(2-pyridy)-imin-кN-N-(2-thiazin-кN-2-y)thiazidine]cbat() nitrate has been isolated and characterized by single crystal X-ray diffraction, IR spectroscopy, UV-Vis-NIR diffuse reflectance and magnetic susceptibility measurements. The environment around the cobalt atom may be described as a distorted octahedral geometry with the ligand-metal-ligand bite angles varying between 84.07(8)^°^ and 98.66(8)^°^.The metallic atom is coordinated to two thiazoline nitrogens [av. Co-N =2.067 Å], two imino nitrogens [av. Co-N =2.122 Å], one oxygen atom of the nitrate group monodentate [Co-O(1)= 2.249(2) Å] and the oxygen atom of the water molecule [Co-O(IW)= 2.105(2) Å]. Electronic UV-Vis-NIR spectral data and the calculated magnetic moment are indicative of octahedral Co(ll) complexes. In the same way as other PyTT complexes, the organic moiety preserves the imino-thiazolidine form detected in the structure of PyTT.


### Synthesis and Characterization of a New Cobalt(II) Complex with 2-(2-Pyridyl)Imino-N-(2-Thiazolin-2-yl)Thiazolidine (PyTT)

A. Bernalte-García*, F.J. García-Barros, F.J. Higes-Rolando, F. Luna-Giles, M.M. Pacheco-Rodríguez and E. Viñuelas-Zahínos

*Departamento de Química Inorgánica, Facultad de Ciencias, Universidad de Extremadura, 06071 Badajoz, Spain*

### GRAPHICAL ABSTRACT

The complex $[Co(NO_3)(PyTT)_2(H_2O)]NO_3$ has been isolated and characterized by single crystal X-ray diffraction, IR spectroscopy, UV-Vis-NIR diffuse reflectance and magnetic susceptibility measurements. The environment around the cobalt atom may be described as a distorted octahedral geometry with the metallic atom coordinated to two thiazoline nitrogens, two imino nitrogens, one oxygen atom of a nitrate group monodentate and the oxygen atom of a water molecule.

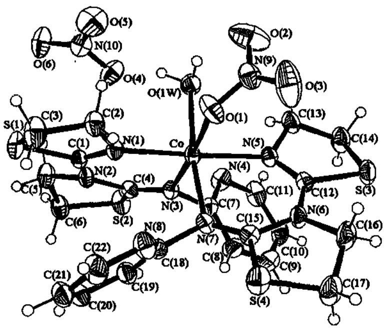

### ABSTRACT

The compound aquanitrate-κ*O*-bis[2-(2-pyridyl)-imino-κ*N*-N-(2-thiazolin-κ*N*-2-yl)thiazolidine]cobalt(II) nitrate has been isolated and characterized by single crystal X-ray diffraction, IR spectroscopy, UV-Vis-NIR diffuse reflectance and magnetic susceptibility measurements. The environment around the cobalt atom may be described as a distorted octahedral geometry with the ligand-metal-ligand bite angles varying between 84.07(8)° and 98.66(8)°. The metallic atom is coordinated to two thiazoline nitrogens [av. Co-N = 2.067 Å],

*Corresponding author. Tel.: + 34-924-289-395; fax: + 34-924-289-394.
E-mail address: alberga@unex.es (A. Bernalte-García)

two imino nitrogens [av. Co-N = 2.122 Å], one oxygen atom of the nitrate group monodentate [Co-O(1) = 2.249(2) Å] and the oxygen atom of the water molecule [Co-O(1W) = 2.105(2) Å]. Electronic UV-Vis-NIR spectral data and the calculated magnetic moment are indicative of octahedral Co(II) complexes. In the same way as other PyTT complexes, the organic moiety preserves the imino-thiazolidine form detected in the structure of PyTT.

**Keywords:** Cobalt(II) complex; Pyridine; Thiazoline; Thiazolidine

### INTRODUCTION

Research interest in thiazoline and thiazolidine derivatives has increased during recent years, due to their biological and pharmaceutical activity. 2-thiazolines have been synthesized for many years and they are especially interesting because this heterocycle can be easily converted into thiazole or penicillin derivatives. A lot of new synthetic compounds have emerged due to investigation for improving their therapeutic effect.

It has been proposed that penicillin inhibits the formation of bacterial cellular wall by acting as a structural analog of the terminal D-alanyl-D-alanine residue of the peptidoglycan strand. It is suggested, therefore, that the transpeptidase would first react with the substrate to form an acyl enzyme intermediate, with the elimination of D-alanine, and that this active intermediate would then react with another strand to form the cross-link and regenerate the enzyme. The penicillin fits the substrate-binding site with the β-lactam ring in the same position as the bond involved in the transpeptidation. It therefore acylates the enzyme, forming a penicilloyl enzyme, and thereby inactivates it /1/. In this mechanism, metallic ions might act as catalysts in the field of the coordination chemistry, because in the formation of substrate-enzyme complex metallic ions might provide all or some binding sites through formation of an intermediate enzyme-metal-substrate type bridge complex.

Likewise, many of these compounds containing thiazoline and thiazolidine rings present antitumoral /2/, anti-HIV /3/ and antiinflammatory activity /4/, as well as being known for their application in the treatment of diabetes /5/. However, the biological activity could be modified or even improved thanks to coordination to metallic ions or atoms.

The synthesis of new siderophore analogues constitutes another example of pharmacological application. Microbial siderophores are relatively low molecular weight compounds synthesized in order to solubilize and transport iron (III) into the procariotes cells in the necessary concentrations /6/. New siderophore analogues are prototypes for the synthesis of chelating agents employed for clinical use /7/. Thus, siderophores have been characterized containing thiazoline rings as desferrithiocin or pyochelin. These compounds have been used in the treatment of acute iron poisoning and in the chronic iron overload resulting from transfusion theraphy of β-thalasemia /8/. Likewise, some siderophores form strong chelates with other metal ions like copper(II), nickel(II), zinc(II) /9/, calcium(II), magnesium(II) and cobalt(II) /6/. The research on new siderophores will conduct to new clinical drugs for certain metal detoxification /10/.

For the reasons given above, in recent years we have been investigating the coordination chemistry of compounds containing these heterocycles /11-13/. As part of this study, we report here the synthesis and characterization of the cobalt(II) complex $[Co(NO_3)(PyTT)_2(H_2O)]NO_3$ [PyTT = 2-(2-pyridyl)imino-N-(2-thiazolin-2-yl)thiazolidine].

### EXPERIMENTAL

### Ligand

The ligand 2-(2-pyridyl)imino-*N*-(2-thiazolin-2-yl)thiazolidine (PyTT) was prepared according to a procedure reported elsewhere /14/ taking into account published modifications /15/ and recrystallized from cyclohexane. *Anal.* Found: C, 49.97; H, 4.58; N, 21.19; S, 24.65. Calc. for $C_{11}H_{12}N_4S_2$: C, 49.94; H, 4.49; N, 21.15; S, 23.65%.

### Preparation of $[Co(NO_3)(PyTT)_2(H_2O)]NO_3$

This was obtained by reacting an ethanol-water solution of $Co(NO_3)_2.6H_2O$ (111 mg, 0.38 mmol) with another ethanol-water solution of PyTT (100 mg, 0.38 mmol). The slow evaporation of the solvent yields red monocrystals suitable for X-ray diffraction (112 mg, 40%). *Anal.* Found: C, 35.60; H, 3.24; N, 18.78; S, 16.88. Calc. for $C_{22}H_{26}CoN_{10}O_7S_4$: C, 36.21; H, 3.59; N, 19.18; S, 17.58%.

### Instrumental procedures

Chemical analyses of carbon, hydrogen, nitrogen and sulphur were performed by means of microanalytical methods using a Leco CHNS-932 microanalyser. A UV-Vis-NIR reflectance spectrum in the 200-1000 nm range was obtained from a pellet of the sample, using a Shimadzu UV-3101 PC spectrophotometer and $BaSO_4$ as a reference. Magnetic measurements were made at room temperature on a magnetometer with pendulum MANICS DSM8, equipped with helium continous-flow cryostat and an electromagnetometer DRUSCH EAF 16 UE. IR spectra were recorded on a Perkin-Elmer FT-IR 1720 spectrophotometer, from KBr pellets in the 4000-370 $cm^{-1}$ range and on a Perkin Elmer FT-IR 1700X spectrophotometer, from polyethylene pellets in the 500-150 $cm^{-1}$ range.

### Crystal structure determinations

Crystal data, data collection and refinement details are given in Table 1. The first 50 frames were recollected at the end of data collection to monitor for decay. Absorption and incident beam corrections were applied using the SADABS program /16/. The structure was solved by direct methods and subsequent Fourier differences using the WINGX package /17/ and refined by full-matrix least-squares. All non-hydrogen atoms were refined anisotropically. The hydrogen atoms were placed in their ideal positions.

**Table 1**

Crystal data and structure refinement parameters for $[Co(NO_3)(PyTT)_2(H_2O)]NO_3$.

| | |
|---|---|
| Empirical formula | $C_{22}H_{26}CoN_{10}O_7S_4$ |
| Formula weight | 729.7 |
| Crystal system | Triclinic |
| Space group | $P\bar{1}$ |
| Crystal shape | Plate |
| Crystal size (mm) | 0.30 x 0.22 x 0.08 |
| Unit cell dimensions | |
| $a$ (Å) | 8.101(2) |
| $b$ (Å) | 9.982(3) |
| $c$ (Å) | 19.879(6) |
| α (°) | 96.005(5) |
| β (°) | 96.028(5) |
| γ (°) | 112.468(5) |
| Cell Volume (Å$^3$) | 1459.0(7) |
| Z | 2 |
| $D_{calc}$ (g cm$^{-3}$) | 1.661 |
| μ (mm$^{-1}$) | 0.936 |
| F(000) | 750 |
| Temperature (K) | 293±2 |
| Diffractometer | Bruker SMART CCD |
| Radiation, wavelength (Å) | Mo Kα, 0.71073 |
| Collection method | φ-ω |
| Absorption correction | Empirical |
| 2θ Range (°) | 4.2-56.7 |
| Index ranges | $-5 \leq h \leq 10$ |
| | $-13 \leq k \leq 12$ |
| | $-25 \leq l \leq 25$ |
| Independent reflections | 6631 |
| Reflections observed [F>4σ(F)] | 4290 |
| Number of refined parameters | 405 |
| R | 0.0409 |
| wR | 0.0825 |
| GOF | 1.013 |
| $\rho_{max}$, $\rho_{min}$ (eÅ$^{-3}$) | 0.307, -0.278 |

### RESULTS AND DISCUSSION

The analytical results agree with the molecular formula $C_{22}H_{26}CoN_{10}O_7S_4$ for the red Co(II) complex.

### Description of the structure

The X-ray study revealed that the crystals of $[Co(NO_3)(PyTT)_2(H_2O)]NO_3$ are made up of triclinic units cells, containing $[6(NO_3)(PyTT)_2(H_2O)]^+$ cations and nitrate anions. Figure 1 shows the molecular structure of the cation complex. The most relevant bond lengths and angles are given in Table 2.

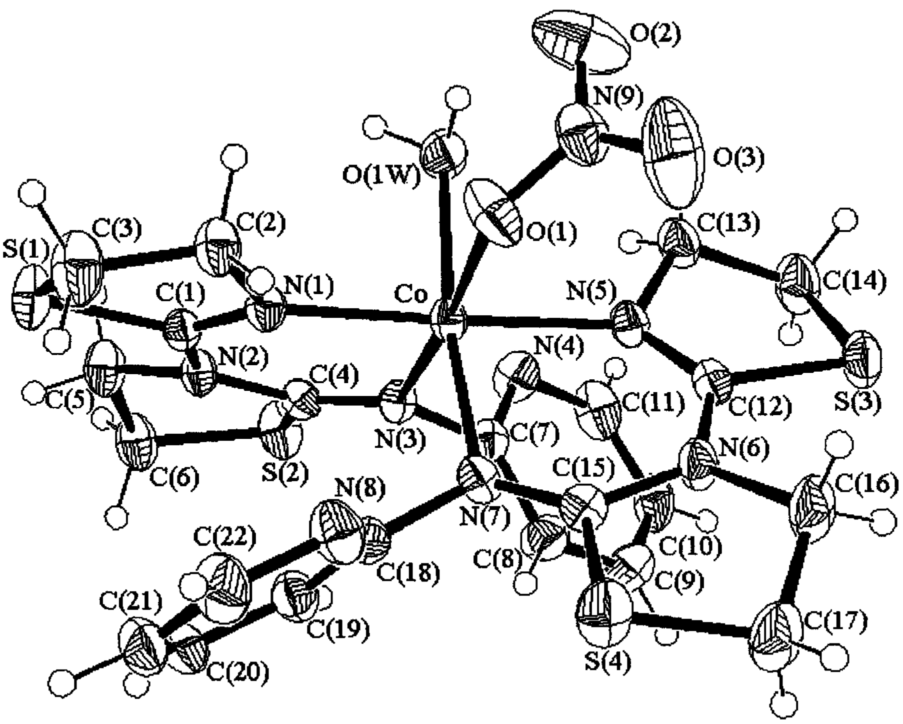

**Fig. 1:** Molecular structure of $[Co(NO_3)(PyTT)_2(H_2O)]^+$ cation, showing the atom-numbering scheme. The thermal ellipsoids are drawn at a 40% level.

The environment around the cobalt(II) atom may be described as a distorted octahedral geometry with the ligand-metal-ligand bite angles varying between 84.07(8)° [N(7)-Co-O(1)] and 98.66(8)° [N(3)-Co-N(7)]. The metallic atom is coordinated by two thiazoline nitrogens [av. Co-N = 2.067 Å], two imino nitrogens [av. Co-N = 2.122 Å], one oxygen atom of the nitrate group monodentate [Co-O(1) = 2.249(2) Å] and the oxygen atom of the water molecule [Co-O(1W) = 2.105(2) Å]. This compound is the second structurally characterized compound having a 2-thiazoline link to cobalt(II) /12/, being, moreover, the first with an octahedral geometry. The Co-O(1W) bond length is similar and comparable to the calculated average value [2.134(99) Å] for 45 crystal structures with an octahedral cromophore group $CoN_4O_2$ obtained from the Cambridge Structural Database System(CSD). Likewise, the Co-N(imino) distances [Co-N(3) = 2.113(2) Å;

Co-N(7) = 2.130(2) Å] are in good agreement with the mean value calculated using the data of 28 crystal structures obtained from CSD. However, the Co-$O_{nitrate}$ bond distance [Co-O(1) = 2.249(2) Å] is longer that the mean value 2.109(40) Å calculated from the data of 33 crystal structures of octahedral Co(II) complexes collected in the CSD.

**Table 2**

Selected bond lengths (Å), angles (°) and hydrogen bonds for $[Co(NO_3)(PyTT)_2(H_2O)]NO_3$

| | | | |
|---|---|---|---|
| *Bond lengths* | | | |
| Co-O(1) | 2.249(2) | Co-O(1W) | 2.105(2) |
| Co-N(1) | 2.059(2) | Co-N(3) | 2.113(2) |
| Co-N(5) | 2.074(2) | Co-N(7) | 2.130(2) |
| C(12)-N(5) | 1.278(3) | C(13)-N(5) | 1.479(3) |
| C(15)-N(6) | 1.389(3) | C(16)-N(6) | 1.475(3) |
| C(12)-N(6) | 1.378(3) | C(18)-N(7) | 1.432(3) |
| C(15)-N(7) | 1.287(3) | C(1)-N(1) | 1.286(3) |
| C(2)-N(1) | 1.474(3) | C(1)-N(2) | 1.380(3) |
| C(4)-N(2) | 1.389(3) | C(5)-N(2) | 1.483(3) |
| C(4)-N(3) | 1.286(3) | C(7)-N(3) | 1.434(3) |
| *Bond angles* | | | |
| O(1W)-Co-N(1) | 90.49(8) | O(1W)-Co-N(3) | 92.31(8) |
| O(1W)-Co-N(5) | 88.66(8) | O(1W)-Co-O(1) | 85.29(9) |
| N(1)-Co-N(3) | 85.38(8) | N(1)-Co-N(7) | 94.91(8) |
| N(1)-Co-O(1) | 90.72(8) | N(3)-Co-N(5) | 97.85(8) |
| N(3)-Co-N(7) | 98.66(8) | N(5)-Co-N(7) | 85.35(8) |
| N(5)-Co-O(1) | 86.03(8) | N(7)-Co-O(1) | 84.07(8) |
| N(1)-C(1)-S(1) | 117.7(2) | N(5)-C(12)-S(3) | 117.5(2) |
| N(1)-C(1)-N(2) | 125.3(2) | N(5)-C(12)-N(6) | 125.9(2) |
| N(3)-C(4)-N(2) | 124.3(2) | N(7)-C(15)-N(6) | 124.2(2) |
| N(3)-C(4)-S(2) | 125.0(2) | N(7)-C(15)-S(4) | 125.1(2) |
| C(4)-N(3)-C(7) | 117.0(2) | C(15)-N(7)-C(18) | 117.1(2) |
| *Bond A...H-D* | *Position of D* | *A...D (Å)* | *A...H-D(°)* |
| O(1W)-H(1W)...O(2) | x, y, z | 2.891(4) | 145(2) |
| O(1W)-H(2W)...O(4) | x, y, z | 2.697(3) | 160(1) |

The six-membered chelate ring Co-N(1)-C(1)-N(2)-C(4)-N(3) shows a conformation near to half-chair, with Co and N(3) deviating 0.134 and 0.144 Å above and below, respectively, the plane formed by N(1)-

C(1)-N(2)-C(4). This geometry is proved by the puckering parameters Q = 0.170 Å, $\phi$ = 329.1° and $\theta$ = 49.7° calculated according to Cremer and Pople /18/. Therefore, the chelate ring Co-N(5)-C(12)-N(6)-C(15)-N(7) presents a conformation near to half-chair [Q = 0.295 Å; $\phi$ = 332.4°; $\theta$ = 58.4°], with Co y N(7) deviating 0.266 and 0.220 Å below and above, respectively, the N(5)-C(12)-C(15)-N(7) mean plane. In the organic ligands, the values of the puckering parameters for the thiazoline and thiazolidine rings calculated according to Cremer and Pople /18/, are:

| | | |
|---|---|---|
| Ring A: [S(1)-C(1)-N(1)-C(2)-C(3)] | $q_2$ = 0.244 Å; | $\phi$ = 318.8°; |
| Ring B: [S(3)-C(12)-N(5)-C(13)-C(14)] | $q_2$ = 0.290 Å; | $\phi$ = 317.2°; |
| Ring C: [S(2)-C(4)-N(2)-C(5)-C(6)] | $q_2$ = 0.347 Å; | $\phi$ = 318.9°; |
| Ring D: [S(4)-C(15)-N(6)-C(16)-C(17)] | $q_2$ = 0.387 Å; | $\phi$ = 324.1°; |

These values indicate that four rings show a conformation near to envelope with the apexes at C(3), C(14), C(6) and C(17), respectively.

As in other PyTT complexes /11,12/, the organic moiety preserves the imino-thiazolidine form detected in the structure of PyTT. However, a drastic change is observed in the conformation upon complexation. Thus, the thiazoline rings are rotated around the C(1)-N(2) and C(12)-N(6) bonds, respectively, which permit the donation through N(1) and N(5) [torsion angles S(1)-C(1)-N(2)-C(5) = 11.9° and S(3)-C(12)-N(6)-C(16) = 13.7° in the complex; 176.5° in PyTT]. Similarly, the rotation angles of the pyridine rings around the C(7)-N(3) bond [torsion angle C(4)-N(3)-C(7)-N(4) = 68.8°] and C(18)-N(7) bond [torsion angle C(15)-N(7)-C(18)-N(8) = 54.0°] are higher than in PyTT (16.9°) which decreases the steric interaction between both ligands of the cation complex.

The presence of hydrogen bonds is observed. Thus, the water molecule of coordination acts as donor of hydrogen, while oxygen atoms O(4) of nitrate ion and O(2) of nitrate ligand monodentate act as acceptors.

### Physical measurements

The observed molar magnetic susceptibility for $[Co(NO_3)(PyTT)_2(H_2O)]NO_3$ was corrected for diamagnetism and temperature-independent paramagnetism to provide the fully corrected magnetic moment at room temperature ($\mu$ = 5.0 BM). This value is consistent with the octahedral environment around cobalt(II) /19/.

The diffuse reflectance spectrum shows one intense band at 31250 $cm^{-1}$, which is indicative of one charge-transfer transition. Moreover, the spectrum exhibits two broad bands with considerable fine structure that have intensities consistent with spin-allowed d-d transitions. These bands may be assigned as: $\nu_1[^4T_{1g}(F) \rightarrow {}^4T_{2g}(F)]$(11340, 8320, 5870 $cm^{-1}$) and $\nu_3[^4T_{1g}(F) \rightarrow {}^4T_{1g}(P)]$(19230 $cm^{-1}$) transitions, in an idealized $O_h$ symmetry. The ligand field parameters, 10 Dq and B, were calculated from the averaged values of the $\nu_1$ and $\nu_3$ bands taking the centre of gravity of the total intensity /20/, using the following known relationships /21/ for octahedral $d^7$ complexes:

$$\nu_1[^4T_{1g}(F) \rightarrow {}^4T_{2g}(F)] = 1/2(10Dq - 15B) + 1/2[(10Dq + 15B)^2 - 12B \cdot 10Dq]^{1/2}$$

$$\nu_3[^4T_{1g}(F) \rightarrow {}^4T_{1g}(P)] = 1/2[(10Dq + 15B) - 12B \cdot 10Dq]^{1/2}$$

The calculated values of the ligand field parameters 10Dq = 9220 $cm^{-1}$ and B = 770 $cm^{-1}$ are in fair agreement with the predicted values for octahedral complexes and are consistent with the presence of a cromophere group $[CoN_4O_2]$ /22-24/.

The IR spectrum in the 4000-370 $cm^{-1}$ region shows the presence of bands due to the thiazoline ring vibrations at 1590 ($W_1$), 947 and 895 ($W_3$), 815 ($W_4$), 754 and 740 ($W_5$), 646 ($W_6$), 533 ($W_7$), 598 ($W_8$), 445 and 434 $cm^{-1}$ ($\Gamma_1$) /25/. Likewise, the spectrum exhibits bands assignable to thiazolidine ring vibrations at 1028 ($\nu_1$), 947 ($\nu_2$), 883 and 839 ($\nu_3$), 704 ($\nu_4$), 687 and 635 ($\nu_5$), 582 and 571 ($\delta_1$), 514 and 495 $cm^{-1}$ ($\delta_2$) /26,27/. Moreover, characteristic bands of pyridine ring vibrations are detected at 1573 (8a), 1551 (8b), 1465 (19a), 1433 (19b), 651 (6b), 624 (6a) and 411 (16b) $cm^{-1}$ /28,29/.

Other relevant bands are registered at 3500 and 3425 ($\nu_{H_2O}$), 1618 [$\nu_{(C=N)imine}$] and 1124 $cm^{-1}$ [$\nu_{(C-N)}$] /30/.

In the 1700-1800 $cm^{-1}$ region of the IR spectrum, the pattern of the $\nu_1+\nu_4$ combination bands was studied. The value of the $\nu_1+\nu_4$ splittings ($\Delta$ = 33 $cm^{-1}$) indicates, according to Lever's criteria /31/, the presence of one monodentate nitrate group. Likewise, a third peak was observed in this region, which allows to recognize a ionic nitrate group. Moreover, the IR spectrum shows other bands at 1288, 1028, 806, 721 and 704 $cm^{-1}$ that can be assigned to the monodentate nitrate group whereas the bands at 1380 and 826 $cm^{-1}$ can be assigned to the nitrate ion group [31-36].

Only four bands assignable to Co-ligand stretching vibrations are observed in the low frequency range, although the $C_1$ symmetry of the complex cation predicts the appearance of six such bands. The broad band at 346 $cm^{-1}$ is assignable to the $\nu(Co-OH_2)$ mode of the coordinated water /22,37,38/, whereas the band at 324 $cm^{-1}$ is assigned to $\nu(Co-N_{imine})$, in good accord with literature /39-42/.

Finally, the bands at 270 and 223 $cm^{-1}$ are tentatively assigned to the $\nu(Co-ONO_2)$ /37,43,44,45/ and to $\nu(Co-N_{thiazoline})$ /12/, respectively.

### CONCLUSIONS

From the structural study of this complex some conclusions can be drawn. First, it can be expected that this complex presents little or no antimicrobial activity due to the fact that the complex is octahedral and presents a complete coordination sphere /46/, which hinders the entry of new ligand to form the complex enzyme-metal-substrate complex. Secondly, since cobalt intoxications induce cardiomyopathy, allergic dermatitis and asthma /47/, it can be possible to use PyTT as siderophore for the treatment of poisoning with this metal due to the formation of a complex with chelate rings.

### SUPPLEMENTARY MATERIAL

CCDC 209791 contains the supplementary crystallographic data for this paper. These data can be obtained free of charge via www.ccdc.cam.ac.uk/conts/retrieving.html (or from the Cambridge Crystallographic Data Centre, 12, Union Road, Cambridge CB2 1EZ, UK; fax: +44 1223 336033; or deposit@ccdc.cam.ac.uk).

### ACKNOWLEDGEMENTS

We would like to thank for financial support the Spanish Ministerio de Ciencia y Tecnología [under the project BQ01-0374], the Junta de Extremadura (Consejería de Educación, Ciencia y Tecnología) and the FEDER [under the projects IPR00A028 and 2PR02A098].

### REFERENCES

1. L. Stryer, *Biochemistry,* W. H. Freeman, New York, 1995.
2. P. Wipf, P. Fritch, *Tetrahedron Lett.*, **35**, 5377 (1994).
3. M. Premanathan, R. Arakaki, S. Ramanan, S. Jinno, M. Baba, S. Yamashita, H. Nakashima, *Antiviral Chemistry & Chemotherapy*, **9**, 423 (1998).
4. C. Della Monica, A Randazzo, G. Bifulco, P. Cimino, M Aquino, I. Izzo, F. De Riccardis, L. Gomez-Paloma, *Tetrahedron Lett.*, **43**, 5707 (2002).
5. M. Fukuzawa, J. Satoh, X. Qiang, S. Miyaguchi, Y. Sakata, T. Nakazawa, F. Ikehata, S. Ohta, T. Toyota, *Diab. Res. Clin. Pract.*, **43**, 147 (1999).
6. E Farkas, E.A. Enyedy, H. Csóka, *Polyhedron*, **18**, 2391 (1999).
7. M.L. Guerinot, *Annu. Rev. Microbil.*, **48**, 743 (1994).
8. R.J. Bergeron, C.Z. Liu, J.S. McManis, M.X.B. Xia, S.E. Algee, J Wiegand, *J. Med. Chem.*, **37**, 1411 (1994).
9. E. Farkas, H Csóka, G. Micera, A. Dessi, *J. Inorg. Biochem.*, **65**, 281 (1997).
10. K. Langemann, D. Heineke, S. Rupprecht, K.N. Raymond, *Inorg. Chem.*, **35**, 5663 (1996).
11. A. Bernalte, F.J.G. Barros, F.J. Higes, F. Luna, *Polyhedron*, **18**, 2907 (1999).
12. A. Bernalte, F.J.G. Barros, F.J. Higes, F. Luna, *Polyhedron*, **20**, 3315 (2001).
13. A. Bernalte, F.J.G. Barros, F.J. Higes, F. Luna, R. Pedrero, *J. Inorg. Biochem.*, **86**, 144 (2001).
14. R.J. Outcalt, *J. Heterocycl. Chem.*, **24**, 1425 (1987).
15. M. Ávalos, R. Babiano, P. Cintas, J.L. Jiménez, J.C. Palacios, C. Valencia, *Heterocycles*, **35**, 1239 (1993).
16. R.H. Blessing, *Acta Crystallogr*, **Sect. A 51**, 33 (1995).
17. L. J. Farrugia, *J. Appl. Cryst.*,**32**, 837 (1999).
18. D. Cremer, J.A. Pople, *J. Am. Chem. Soc.*, **97**, 1354 (1975).
19. B.N. Figgis, J. Lewis, *Prog. Inorg. Chem.*, **6**, 185 (1967).
20. F.A. Cotton, D.M.L. Goodgame, M. Goodgame, *J. Am. Chem. Soc.*, **83**, 4690 (1961).
21. D.K. Rastogi, K.C. Sharma, S.K. Dua, M.P. Teotia, *J. Inorg. Nucl. Chem.*, **37**, 685 (1975).
22. G. Alzuet, S. Ferrer, J. Borrás, A. Castiñeiras, X. Solans, M. Font-Bardía, *Polyhedron*, **11**, 2849 (1992).
23. W.U. Malik, R. Bembi, R. Singh, *Polyhedron*, **2**, 369 (1983).
24. J. Dillen, A.T.H. Lenstra, J.G. Haasnoot, J. Reedijk, *Polyhedron*, **2**, 195 (1983).
25. G. Mille, J.L. Meyer, J. Chouteau, *J. Mol. Struct.*, **50**, 247 (1978).
26. M. Guiliano, G. Mille, J. Chouteau, *J. Mol. Struct.*, **50**, 233 (1978).

27. M. Guiliano, G. Mille, T. Avignon, J. Chouteau, *J. Raman Spectrosc.*, 7, 214 (1978).
28. L. Corrsin, B.J. Fax, R.C. Lord, *J. Chem. Phys*, **21**, 1170 (1953).
29. G.L. Cook, F.M. Church, *J. Chem. Phys*, **61**, 458 (1957).
30. L.J. Bellamy, *The Infrared Spectra of Complex Molecules*, Chapman and Hall, London, 1975.
31. A.B.P. Lever, E. Mantovani, B.S. Ramaswamy, *Can. J. Chem.,* **49**, 1957 (1971).
32. N.F. Curtis, Y.M. Curtis, *Inorg. Chem.*, **4**, 804 (1965).
33. B.N. Gaterhouse, S.E. Livingstone, R.S. Nyholm, *J. Chem. Soc.*, 4222 (1957).
34. J.W.F.M. Schoonhoven, W.L. Driessen, J. Reedijk, G.C. Verschoor, *J. Chem. Soc., Dalton Trans.*, **1984**, 1053.
35. G.L. Kleywegt, W.G.R. Wiesmeijer, G.J. Van Driel, W.L. Driessen, J. Reedijk, J.H. Noordik, *J. Chem.. Soc., Dalton Trans.*, **1985**, 2177.
36. M. Goldstein, R.J. Huches, *Inorg. Chim. Acta*, **37**, 71 (1979).
37. R. Battistuzzi, *Polyhedron*, **4**, 993 (1985).
38. J.M. Salas, C. Enrique, M.A. Romero, K. Tacagi, K. Aoki, Y. Miyashita, I. Suh, *Polyhedron*, **11**, 2903 (1992).
39. B. Sing, R.N. Sing., R.C. Aggarwal, *Polyhedron*, **4**, 401 (1985).
40. N.K. Sing, S.Agrawal, R.C. Aggarwal, *Polyhedron*, **3**, 1271 (1984).
41. M. Sahkir, S.P. Varkey, F. Firdaus, P.S. Hamed, *Polyhedron*, **13**, 2319 (1994).
42. M.M. Mostafa, M.A. Khattab, K.M. Abrahim, *Polyhedron*, **2**, 583 (1983).
43. D.X. West, S. Hulslander, *J. Inorg. Chem.*, **43**, 947 (1981).
44. G. Kanagaraj, G.N. Rao, *Polyhedron*, **12**, 383 (1993).
45. W.U. Malik, R. Bembi, R. Singh, *Polyhedron*, **2**, 369 (1983).
46. M.B. Ferrari, F. Bisceglie, G. Pelosi, P Tarasconi, R. Albertini, A. Bonati, P. Lunghi, S. Pinelli, *J. Inorg. Biochem.*, **83**, 169 (2001).
47. O. Andersen, *Chem. Rev.*, **99**, 2683 (1999).

